# Photocatalytic efficacy of supported tetrazine on MgZnO nanoparticles for the heterogeneous photodegradation of methylene blue and ciprofloxacin

**DOI:** 10.1039/c9ra04702f

**Published:** 2019-07-31

**Authors:** Elham Parvizi, Reza Tayebee, Ehsan Koushki, Mojtaba Fattahi Abdizadeh, Behrooz Maleki, Pierre Audebert, Laurent Galmiche

**Affiliations:** Department of Chemistry, School of Sciences, Hakim Sabzevari University Sabzevar 96179-76487 Iran rtayebee@hsu.ac.ir; Department of Physics, School of Sciences, Hakim Sabzevari University Sabzevar 96179-76487 Iran; Cellular and Molecular Research Center, Sabzevar University of Medical Sciences Sabzevar Iran mojtabafattahi@gmail.com; Laboratoire de Photophysique et Photochimie Supramoléculaires et Macromoléculaires, ENS Paris Saclay Cachan France

## Abstract

MgZnO@SiO_2_-tetrazine nanoparticles were synthesized and their photocatalytic efficiency was demonstrated in the decomposition of ciprofloxacin and methylene blue (MB). This new heterogeneous nanocatalyst was characterized by FT-IR, XRD, UV-vis, DRS, FE-SEM, ICP, and CHN. Distinctive variables including photocatalyst dose, pH, and degradation time were investigated. Up to 95% photodegradation was gained under the optimum conditions (20 mg photocatalyst, 3.5 ppm MB, pH 9) by using MgZnO@SiO_2_-tetrazine nanoparticles after 20 min. An elementary kinetic study was carried out, and a pseudo-first-order kinetic with a reasonably high rate-constant (0.068 min^−1^) was derived for the MB decay. Photoluminescence (PL) studies confirmed that the photocatalytic activity of MgZnO@SiO_2_-tetrazine was almost consistent with the Taugh plots. Thus, it can be envisaged that the photocatalytic activity is closely related to the optical absorption. Furthermore, a photoreduction mechanism was suggested for the degradation process. Addition of scavengers and some mechanistic studies also revealed that O_2_˙^−^ is the original radical accounting for the degradation of MB, considering this latter compound as a model type pollutant. Finally, efficacy of the present photocatalytic process was assessed in the degradation of ciprofloxacin as a model drug under the optimum reaction conditions.

## Abstract 2

MgZnO@SiO_2_-tetrazine nanoparticle was synthesized and its photocatalytic efficiency was demonstrated in the decomposition of methylene blue (MB) and ciprofloxacin.

## Graphical abstract

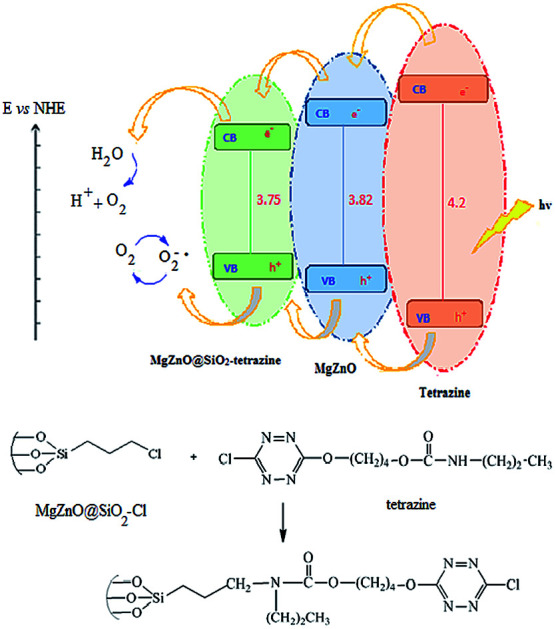

## 1. Introduction

Photocatalytic destruction of organic contaminants with semiconductor-based nanomaterials has attracted substantial attention, because it offers a plausible solution to environmental pollution issues.^1,2^ It is well-known that absorption of photons with an energy higher or equal to the band gap of semi-conductors can promote electrons from the valence to conduction bands and induces a large number of holes in the valence shell. If this charge segregation is valid, the generated holes and electrons can be utilized for the photocatalytic degradation of various organic contaminants. The produced holes are eligible to react with the surface-bound water molecules to generate strong oxidizing species such as the hydroxyl radical. Furthermore, the conduction band electrons might be absorbed by the dissolved molecular oxygen and form superoxide anion-radicals, which can behave as a strong oxidizing agent for most organic compounds.^3,4^ Nowadays, many strategies have been introduced to modify the photocatalytic efficiency of a wide range of semi-conductors by improving the host structure with various metallic and/or organic dopants.^5–9^ Various parameters such as dopant concentration, electronic structure of the semi-conductor, electronic configuration of dopant, and intensity of the illuminating source can strongly affect the photocatalytic efficiency of a system.^10,11^ Moreover, the electronic features of a semi-conductor can be significantly influenced by the dopant characteristics. For example, the photoenergy threshold of a semi-conductor can change and recombination of the photoinduced charge carriers can occur by doping.^12^ It is worth noting that the absence of localized *d*-electrons in the alkaline earth metals mark them as good candidates to alter the photocatalytic properties of a semi-conductor.

Among various semi-conductors, ZnO has been suggested as a perfect photocatalyst for decomposition of water pollutants. Development of the photocatalytic activity of ZnO nanoparticles with metal doping and combination of this material with organic modifiers has already attracted much consideration.^13–17^ Among different organic modifiers, tetrazine ring^18^ which is a very small organic fluorophore with distinct electrochemical and photochemical properties, is selected to be grafted on the surface of ZnO nanoparticles doped with Mg in this report. The tetrazine ring is strongly electron deficient and can be reversibly reduced into its radical anion at a high reduction potential.^19,20^ Therefore, the photocatalytic efficiency of MgZnO nanoparticles can be modified by the surface modification of MgZnO with 4-((6-chloro-1,2,4,5-tetrazine-3yl)oxy)butyl propylcarbamate (abbreviated as tetrazine). The obtained nanocatalyst was used in the photodegradation of MB and ciprofloxacin and effects of some operational variables are investigated.

## 2. Results and discussion

### 2.1. Characterization and properties of MgZnO@SiO_2_-tetrazine

#### 2.1.1. FT-IR spectroscopy

**Fig. 1:**
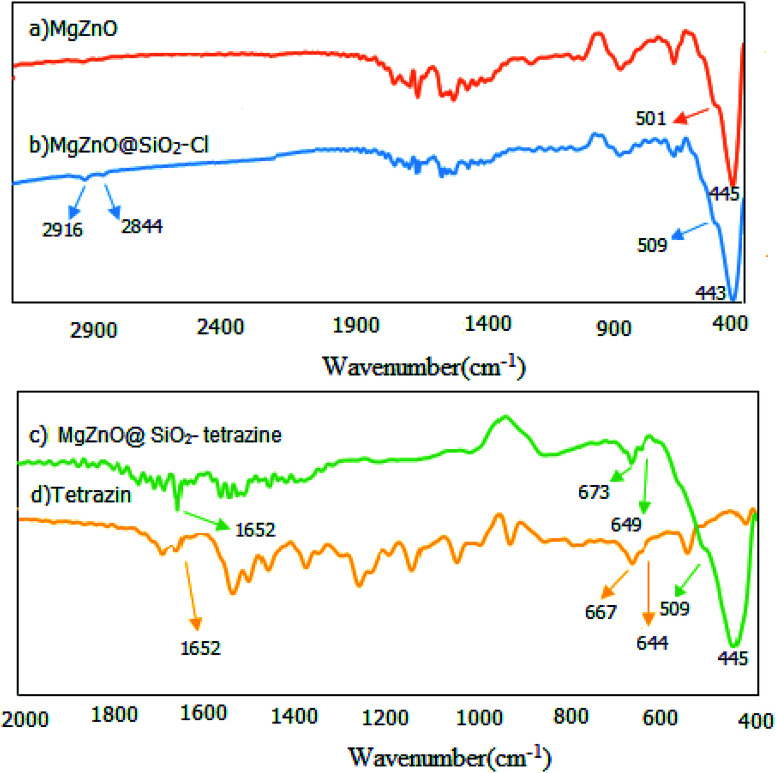
FT-IR spectra of (a) MgZnO, (b) MgZnO@SiO_2_–Cl, (c) MgZnO@SiO_2_-tetrazine, and (d) tetrazine.

The fabricated nanomaterials were characterized by FT-IR spectroscopy. FT-IR spectra of the prepared samples involving MgZnO, MgZnO@SiO_2_–Cl, and MgZnO@SiO_2_-tetrazine are shown in Fig. 1. This study designated the covalent binding and anchoring of tetrazine onto the surface of MgZnO@SiO_2_–Cl. The exceptional absorption bands in the domain of 400–600 cm^−1^ are specific for ZnO. The FT-IR of MgZnO comprised a powerful absorption band at 445 cm^−1^ and a shoulder at 501 cm^−1^ which are due to Zn–O stretching vibrations. Albeit, a small amount of the surface segregated MgO had approximately no apparent influence on the infrared optical properties of ZnO.^21^ The MgZnO was functionalized with CPTS in refluxing toluene to obtain MgZnO@CPTS. Grafting of tetrazine onto the surface of MgZnO@CPTS was approved by the observation of the aliphatic C–H bands around 2844 and 2916 cm^−1^ in the FT-IR spectrum.^22^ Appearance of tetrazine characteristic bands in the FT-IR spectrum of MgZnO@SiO_2_-tetrazine confirmed that the structure of tetrazine was unchanged after immobilization. Observation of weak bands at 1652, 673 and 649 cm^−1^ confirmed grafting of tetrazine moiety onto the surface of MgZnO. However, small shifts in Fig. 1c compared to 1d, would be due to the interaction of tetrazine with the surface functional groups of the solid support.

#### 2.1.2. CHN, ICP, FESEM, and EDX of MgZnO@SiO_2_-tetrazine

**Fig. 2:**
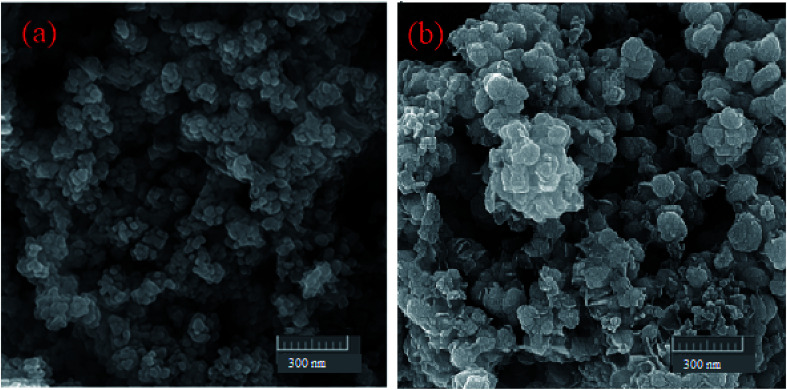
The FE-SEM images of (a) MgZnO and (b) MgZnO@SiO_2_-tetrazine nanoparticles.

**Fig. 3:**
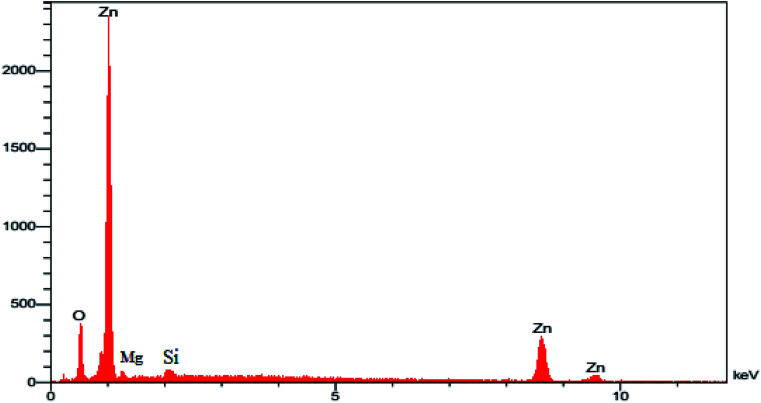
The EDX image of MgZnO@SiO_2_ nanoparticles.

The CHN analysis of MgZnO@SiO_2_-tetrazine revealed the contents of C, 1.78%; H, 0.29%; N, 0.69%, which pointed to the amount of 2.84% for the loaded tetrazine. Moreover, Mg content of MgZnO@SiO_2_-tetrazine sample obtained from ICP analysis was 6.736%. The surface morphology of MgZnO and MgZnO@SiO_2_-tetrazine nanoparticles was further studied by FE-SEM (Fig. 2) and showed disordered irregular nanoscale semi-spherical aggregates of nearly 50 nm in size with a narrow size distribution. It seems that, functionalization with CPTS and anchoring of tetrazine had no significant impacts on the microstructure of MgZnO. EDX analysis confirmed the coexistence of Mg, O, Si, and Zn in MgZnO@SiO_2_ nanoparticles (Fig. 3).

#### 2.1.3. XRD

**Fig. 4:**
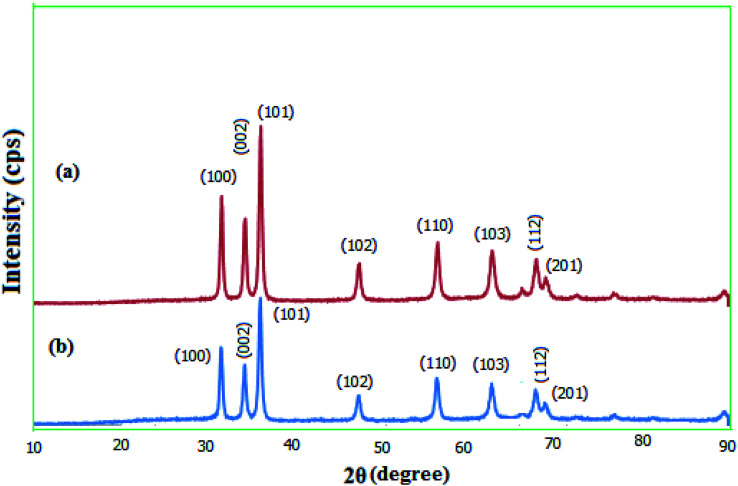
Wide-angle XRD patterns of (a) MgZnO and (b) MgZnO@SiO_2_-tetrazine nanoparticles.

The crystallinity of the prepared MgZnO@SiO_2_-tetrazine nanoparticles was explored *via* wide-angle XRD (Fig. 4). The diffraction patterns for the as-synthesized MgZnO and MgZnO@SiO_2_-tetrazine nanoparticles showed that both materials are nanocrystalline. XRD data matched well with the standard data for the ZnO nanostructure. Similarly, low-intensity peaks at 2*θ* of 31.72, 34.45, 36.21, 47.56, 56.58, 62.89, 67.90, and 69.00 were corresponded to (100), (002), (101), (102), (110), (103), (112), and (201) planes, respectively. These results confirmed that MgZnO@SiO_2_-tetrazine nanoparticles were topologically close to MgZnO.^23^ The approximately unaltered XRD templates for MgZnO@SiO_2_-tetrazine and MgZnO nanoparticles established that no apparent loss of crystallinity was detected after tetrazine grafting. However, rare and small variations in the Bragg intensities were observed.

#### 2.1.4. Photoluminescence (PL) and diffuse spread reflectance (DRS) spectra

**Fig. 5:**
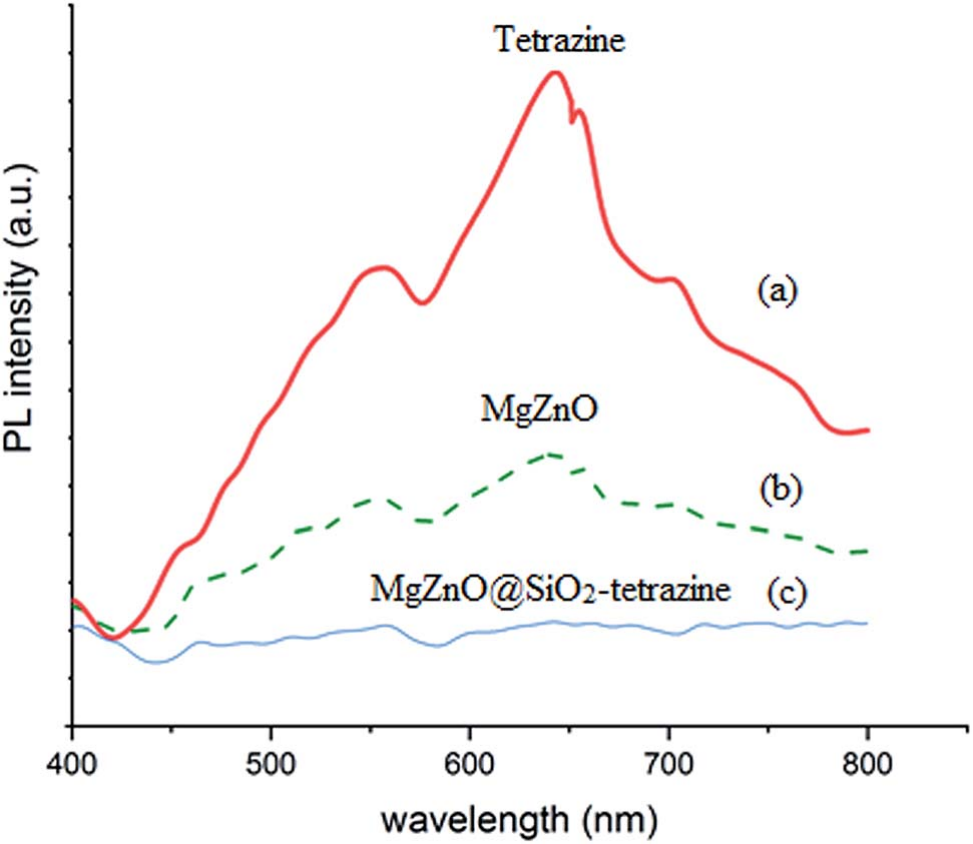
Photoluminescence spectra of tetrazine (a), MgZnO (b), and MgZnO@SiO_2_-tetrazine (c).

PL spectroscopy can be regarded as a powerful technique to characterize the recombination behaviors of the photogenerated electron–hole pairs. The photoluminescence (PL) spectra of the samples are shown in Fig. 5. A UV lamp with the wavelength of 385 nm was used to excite the samples in this experiment. The samples exhibited a broad emission in the wavelength domain of 400–480 nm. Compared to tetrazine and MgZnO, the PL response for MgZnO@SiO_2_-tetrazine showed the strongest emission, suggesting a greatly enhanced radiative recombination of the photogenerated carriers. This result can be attributed to the reduced separation of charge carriers due to formation of the heterojunctions between MgZnO and tetrazine.

**Fig. 6:**
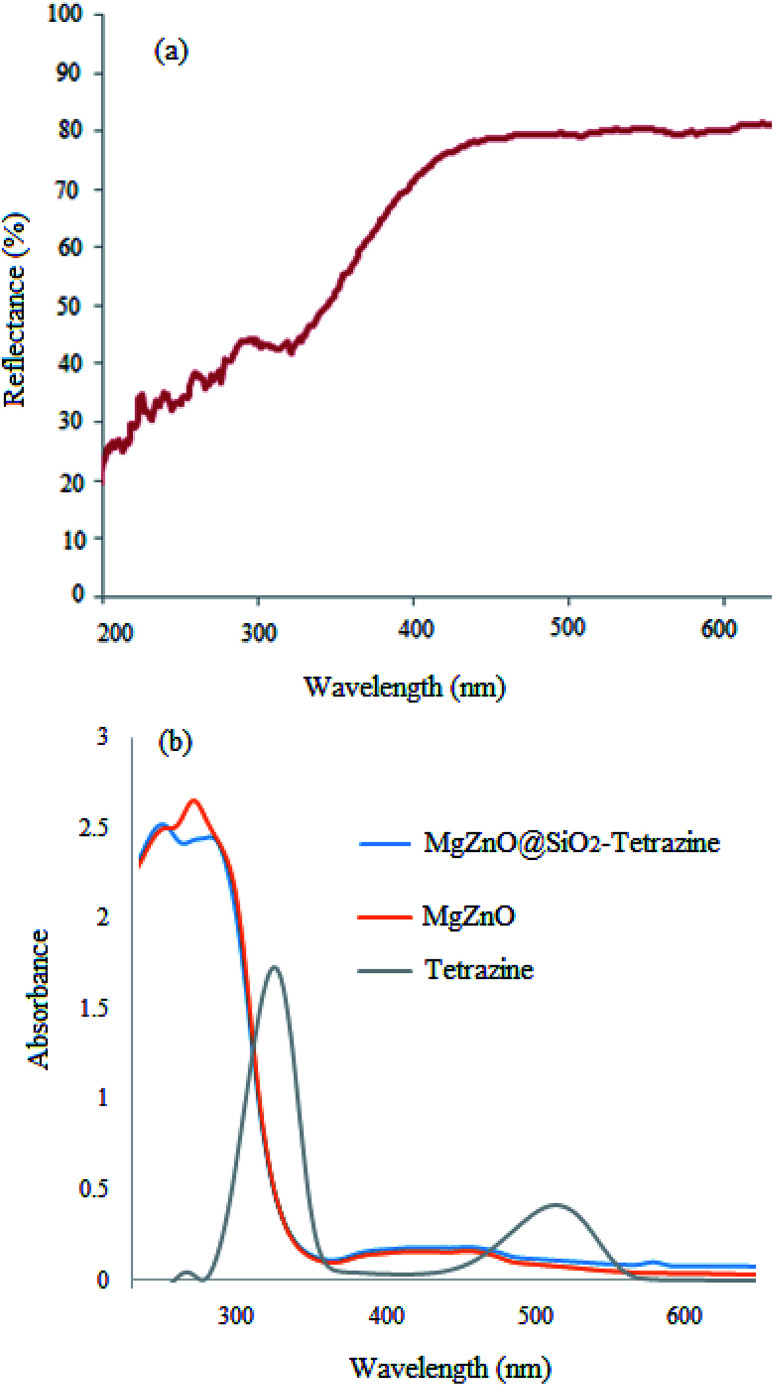
DRS spectrum of MgZnO@SiO_2_-tetrazine (a); UV-vis spectra of MgZnO@SiO_2_-tetrazine, MgZnO, and tetrazine (b).

Optical properties of MgZnO@SiO_2_-tetrazine were also examined by UV-vis diffuse reflectance (DRS) at room temperature (Fig. 6a). The high visible light absorbance demonstrated that MgZnO@SiO_2_-tetrazine nanoparticles would have high visible-light utilization efficiency. As a result of tetrazine anchoring to the surface of MgZnO, the visible-light absorption edge was slightly shifted from ∼425 nm for MgZnO^24^ to about 405 nm, may be due to the presence of tetrazine. Moreover, the UV-vis spectra of MgZnO@tetrazine, MgZnO, and tetrazine are also shown in Fig. 6b.

#### 2.1.5. TGA analysis

**Fig. 7:**
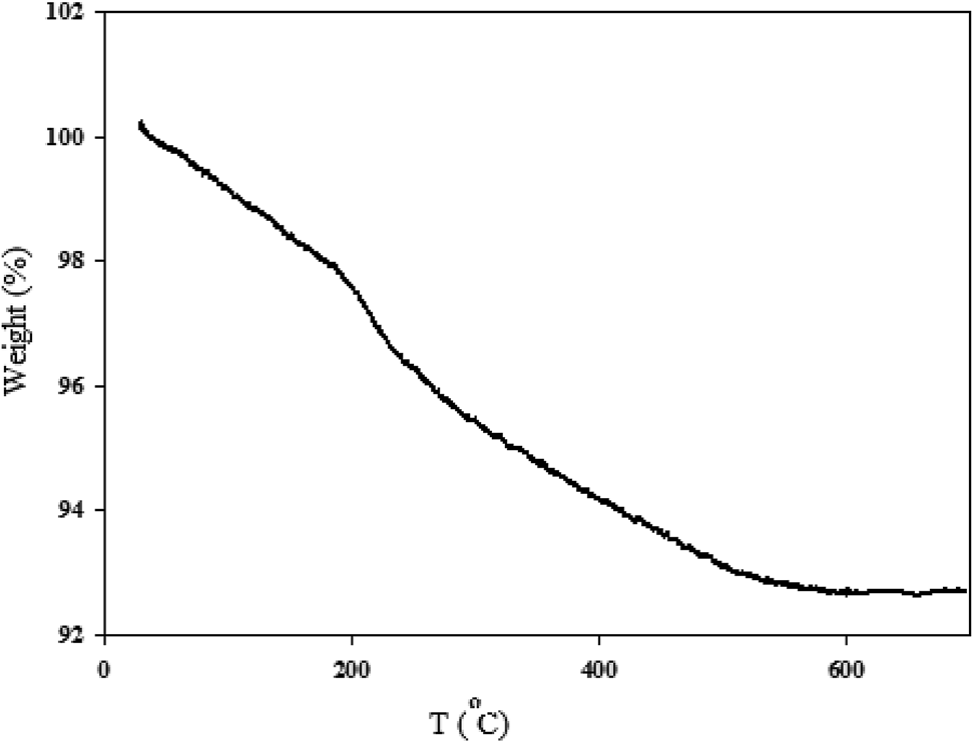
TGA profile of MgZnO@SiO_2_-tetrazine nanoparticles.

The thermal stability of MgZnO@SiO_2_-tetrazine nanocatalyst was investigated by carrying out TGA. As shown in Fig. 7, the sample exhibited three stages of decomposition. The first gradual and continuous weight-loss occurred below 200 °C, which was most likely due to water desorption from the surface of particles and loss of moisture content. Then, tetrazine moiety was started destruction at about 210°. Further loss at higher temperatures was probably corresponded to the progressive burning of the alkyl remnants which was accompanied with the crystallization of zinc oxide up to 530 °C.^25,26^

### 2.2. Photocatalytic activity of MgZnO@SiO_2_-tetrazine

**Fig. 8:**
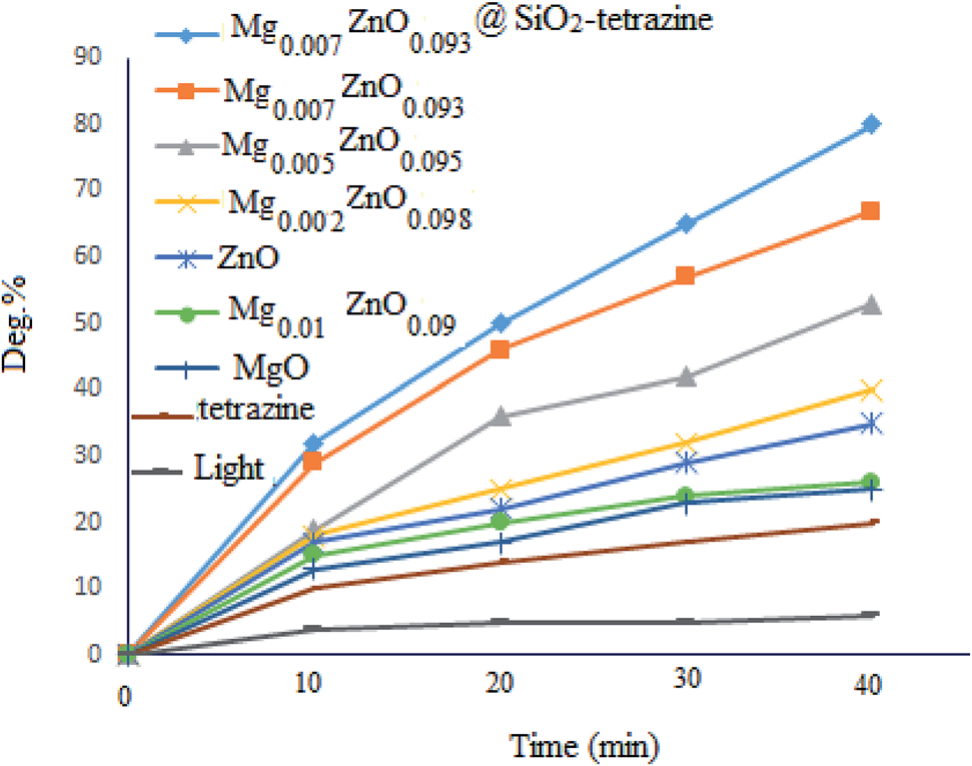
Effects of light and photocatalyst type on the degradation of an aqueous solution of MB ([MB]: 4 ppm, degradation time: 40 min, pH 7).

To evaluate the photocatalytic activity of MgZnO@SiO_2_-tetrazine, photodegradation of MB was picked up in the absence of catalyst and <10% discoloration was reached after 40 min irradiation; whereas, after addition of MgZnO@SiO_2_-tetrazine, the degradation efficiency was clearly increased. However, no significant degradation was attained without irradiation, even, in the presence of MgZnO@SiO_2_-tetrazine (16% degradation after 30 min). The bare tetrazine moiety was almost ineffective even in the presence of light. Some preliminary experiments were achieved to study of the photocatalytic efficiency of various catalysts for the decolorization of an aqueous solution of MB, and the results are detailed in Fig. 8. Before each run, the surface adsorption of MB was calculated in the dark.

According to the above results, a sufficient amount of the photocatalyst (5 mg) was added to a 40 mL solution of MB (4 ppm) and the suspension was stirred for 30 min before sampling. Absorbance of the solution before and after adsorption was applied to determine the surface adsorbed dye. Finally, the best discoloration efficiency was achieved with MgZnO@SiO_2_-tetrazine.

Previous DFT calculations confirmed that the photocatalytic activity of Zn_1−*x*_Mg_*x*_O is highly dependent on the Mg content. Substitution of Mg ions at Zn sites, shifts the conduction band to higher energies and enhances the photocatalytic activity; while, incorporation of Mg^2+^ at the interstitial sites, diminishes the photocatalytic activity.^27^ Therefore, the effects of Mg^2+^ doping on the photocatalytic degradation of MB were also explored. The order of photocatalytic activity as Mg_0.007_Zn_0.093_O > Mg_0.005_Zn_0.095_O > Mg_0.002_ Zn_0.098_O > ZnO > Mg_0.01_Zn_0.09_O > MgO > tetrazine was clearly attained. Thus, balance of the competing doping from lattice substitution and interstitial occupation may explain the optimized photocatalytic activity of MgZnO.

#### 2.2.1. Effect of initial dye concentration

**Fig. 9:**
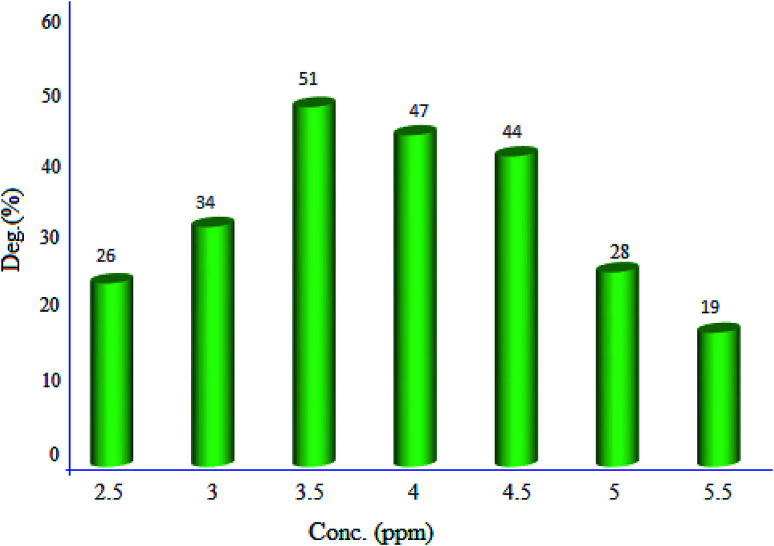
Effect of MB concentration on the discoloration efficiency (40 mL of MB solution, 5 mg of MgZnO@SiO_2_-tetrazine, degradation time 20 min, and pH 7).

Effect of the primary dye concentration (2.5, 3, 3.5, 4, 4.5, 5 and 5.5 ppm) was studied on the photodecolorization process in the presence of MgZnO@SiO_2_-tetrazine (Fig. 9). As shown, an optimum concentration of 3.5 ppm was determined for efficient discoloration of MB. Presumably, at low MB concentration, less dye molecules can approach the catalyst surface, where the hydroxyl radicals are present, and the produced radicals will be deactivated before reaction with the dye molecules, leading to decreasing of the discoloration efficiency.^28^ Thus, with enhancing dye concentration to 3.5 ppm, more MB molecules reacted with the produced hydroxyl radicals; thus, the discoloration was grew up. Under high concentration of MB, dye molecules acted as a filter for the incident light and did not permit the desired light intensity to reach the semi-conductor surface within a limited time lapse; therefore, the photodiscoloration had been decreased.^29^ At this condition, more dye molecules were adsorbed onto the surface of photocatalyst and the active sites of the catalyst would be reduced. Hence, the number of accommodated substrate ions in the interlayer spacing was increased and deactivation of the photocatalyst occurred. Thereby, the amount of reactive O^2−^ and OH^−^ free radicals attacking dye molecules was decreased and lower photodecolorization efficiency was observed.^28,29^

#### 2.2.2. Effect of photocatalyst amount

**Fig. 10:**
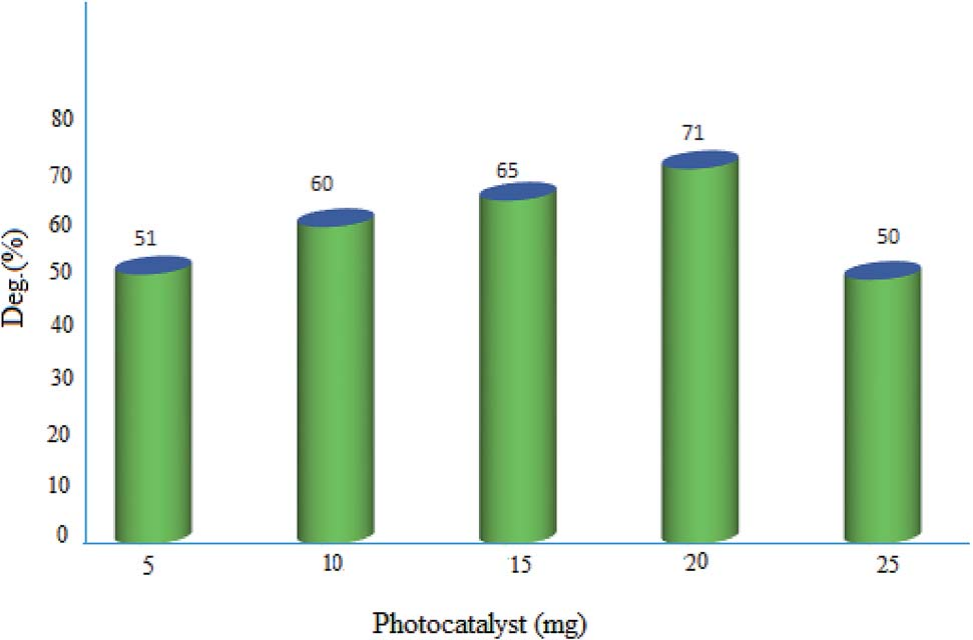
Effect of photocatalyst dosage on the decolorization efficiency of 40 mL solution of dye, [MB]: 3.5 ppm, degradation time: 20 min, and pH 7.

The influence of photocatalyst dosage on the degradation of MB was monitored in the range of 5–25 mg. The represented results in Fig. 10, demonstrated the best decomposition in the presence of 20 mg of MgZnO@SiO_2_-tetrazine nanoparticles. This observation can be explained by the fact that with enhancing the catalyst amount, more active MgZnO@SiO_2_-tetrazine centers would be existed to receive photons and produce hole–electron pairs.^30^ Also, by increasing of the catalyst dosage, the catalyst surface area, light absorption, and the number of active species were increased, hence, degradation of dye was inclined.^31^ Nevertheless, at higher doses outside the optimum value, the solid particles can block penetration of photons; therefore, the overall number of photons approaching the catalyst surface to generate radicals was declined.^32,33^ Moreover, some parts of the catalyst may appear in the dark part and cause diminishing of the light penetration. Inactivation of the activated molecules by collision with ground state molecules, light scattering, screening effects, and aggregation of nanoparticles may also reduce the photocatalytic activity under a high catalyst concentration.

#### 2.2.3. Effect of pH

**Fig. 11:**
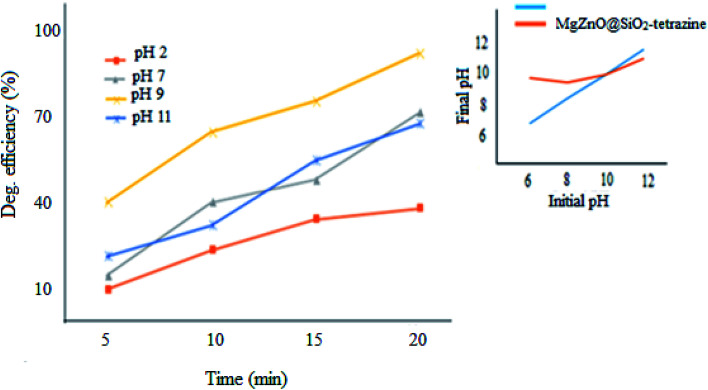
Effect of pH on the photodecolorization efficiency (40 mL of MB solution, MgZnO@SiO_2_-tetrazine: 20 mg, [MB]: 3.5 ppm, degradation time: 20 min).

pH Plays a significant role in the photodegradation efficiency.^34^ Therefore, pH of a series of 3.5 ppm aqueous solutions of MB containing 20 mg of nanocatalyst was changed from 2.0–11.0 and degradation extent of MB was monitored (Fig. 11). The pH_pzc_ for pure ZnO was provided at ∼9; whereas, the value of 10 was attained for MgZnO@SiO_2_-tetrazine.^35^ The surface of MgZnO@SiO_2_-tetrazine has negative and positive charges at pHs higher and lower than pH_pzc_, respectively. Therefore, in strongly acidic conditions, the protonated MB was repelled from the positively charged catalyst surface and degradation was reduced. Thus, with increasing of pH toward pH_pzc_, MB molecules became deprotonated and the catalyst surface felt less charge; hence, more degradation was developed. Moreover, in strongly acidic condition, contamination of the solution with chloride anions from HCl increased concentration of OCl˙ by the reaction of Cl^−^ with OH˙. Thus, considering the lower reactivity of OCl˙ compared to OH˙, degradation of MB was alleviated in strong acidic conditions.^36^ Furthermore, the supported ZnO may be dissolved in a strong acidic pH and diminish the photodegradation efficiency.^33^ At strong basic pHs, free–electron pairs of nitrogen atoms in MB molecules can be repelled from the negatively charged catalyst surface and diminish degradation efficiency. Our findings proved that at pH 9, this repulsion is minimum and a sufficient amount of hydroxyl anions can be generated to produce hydroxyl radicals; so, the best degradation had been observed under this pH.

### 2.3. Reusability and recovery

**Fig. 12:**
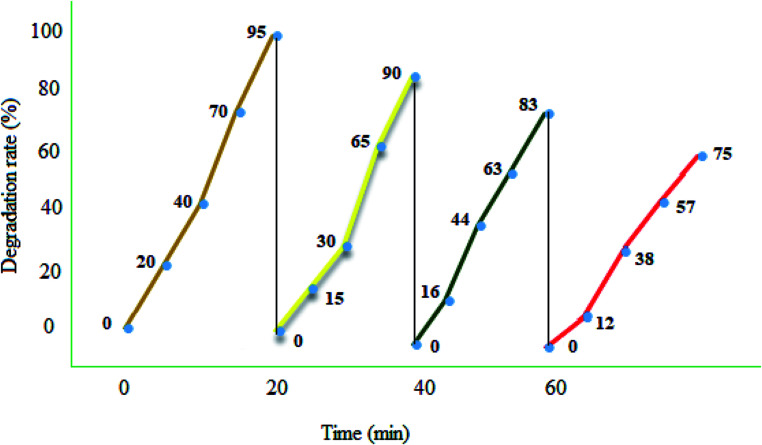
Reusability of MgZnO@SiO_2_-tetrazine in the photodegradation of MB. 40 mL solution of 3.5 ppm MB, MgZnO@SiO_2_-tetrazine: 0.02 g, degradation time: 20 min, and pH 9.

Photocatalyst reuse is a privileged way to assess the ultimate cost of a photocatalytic path. The catalytic stability of the recycled MgZnO@SiO_2_-tetrazine nanoparticles was investigated *via* degrading MB under the optimum conditions over four consecutive runs. A simple proposed treatment flow chart for the reusability of MgZnO@SiO_2_-tetrazine is shown in Fig. 12. Degradation of MB was fulfilled at 25 °C for 20 min in the attendance of MgZnO@SiO_2_-tetrazine. 95% Degradation was achieved at this stage. After that, the nanocatalyst was separated off and the reaction was continued with the filtrate for another 20 min under analogous reaction conditions. Only, 5% reduction in degradation was observed. This result demonstrated no significant release of the catalyst components (principally tetrazine) during the reaction. After 4^th^ run, a smooth reduction in the photocatalytic activity was observed and degradation reached to 75%, may be due to the loss of reused catalyst during each time sampling, irreversible changes of the photocatalyst surface by pollutants, and probable leaking of the photoactive tetrazine moiety from the surface of MgZnO.

### 2.4. Photodegradation of ciprofloxacin as a model drug with MgZnO@SiO_2_-tetrazine

**Fig. 13:**
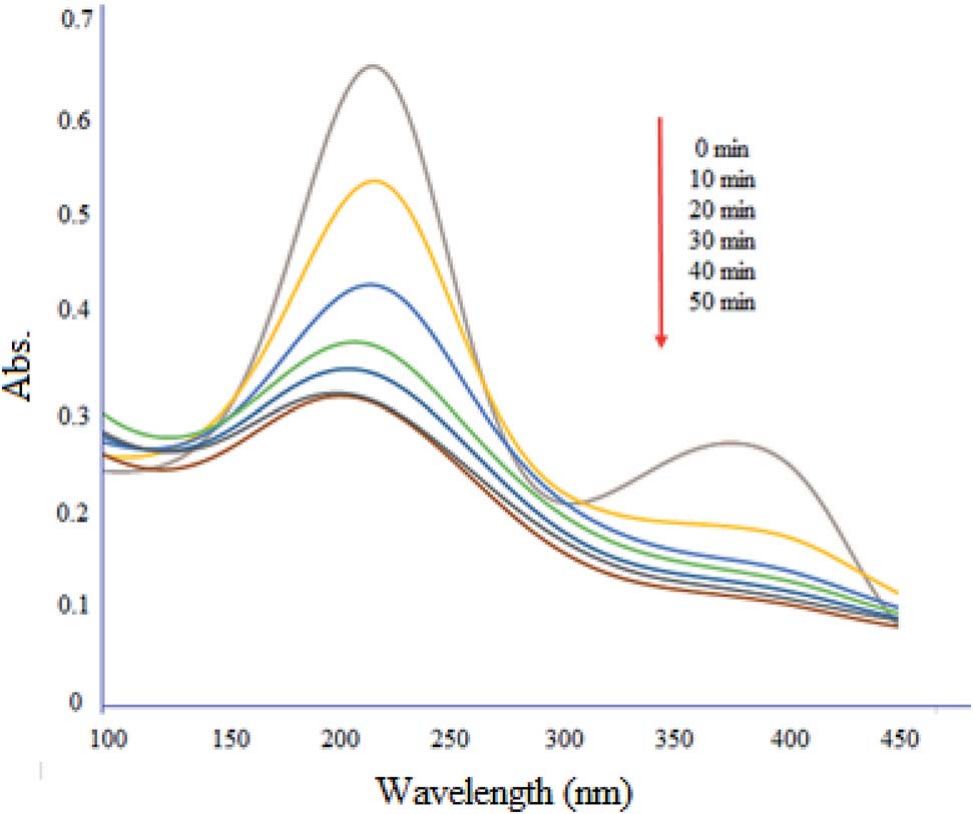
Degradation of ciprofloxacin with time in the presence of MgZnO@SiO_2_-tetrazine.

Fig. 13 shows degradation profile of ciprofloxacin (10 mg L^−1^) by MgZnO@SiO_2_-tetrazine under UV-vis light irradiation. The absorption maximum of the drug was 50% decreased in the presence of light after 50 min. Furthermore, the absence of new peaks during the degradation process, indicated that MgZnO@SiO_2_-tetrazine did not trigger the emergence of new contaminants.^37^

### 2.5. A brief kinetic study of MB degradation

**Fig. 14:**
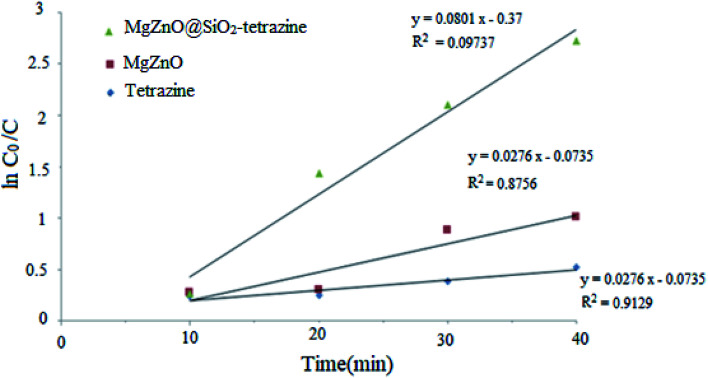
Kinetics of MB degradation catalyzed by tetrazine, MgZnO, and MgZnO@SiO_2_-tetrazine (40 mL of 3.5 ppm MB solution, photocatalyst: 20 mg, degradation time: 20 min, and pH 9).

**Fig. 15:**
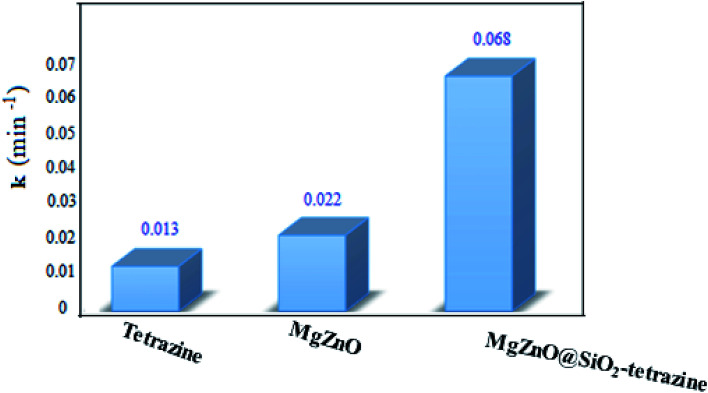
Reaction kinetic rate constants for MB photodegradation over tetrazine, MgZnO, and MgZnO@SiO_2_-tetrazine.

A kinetic study was planned for the photocatalytic degradation of MB with MgZnO@SiO_2_-tetrazine as a function of time. As Fig. 14 shows, a pseudo-first-order kinetic was developed for MB degradation according to the Langmuir–Hinshelwood model.^32^ The photodegradation rate of MB can be shown by eqn (1):

1 ln(*C* − *C*_0_) = −*kt*

where *C*_0_ is the initial concentration of MB (mg L^−1^), *C* is the MB concentration after irradiation, *k* is the rate constant calculated from the slope of the straight line portion of the plot of log(*C*/*C*_0_) *versus t*. The lower photocatalytic activity of tetrazine compared to MgZnO@SiO_2_-tetrazine would be explained by fast recombination of electrons and holes that are generated under the light illumination. Grafting of tetrazine over MgZnO modified the charge separation and the photocatalytic activity of tetrazine was increased. Furthermore, dispersion of tetrazine onto the surface of MgZnO nanoparticles raised the number of active sites for the photoreduction of MB. According to Fig. 15, the rate constants, *k*, of 0.013, 0.022, and 0.068 min^−1^ were calculated for bare tetrazine, MgZnO, and MgZnO@SiO_2_-tetrazine, respectively.

### 2.6. Mechanistic aspects for the photodegradation of MB with MgZnO@SiO_2_-tetrazine

#### 2.6.1. Effect of scavengers

When, holes and electrons get away from recombination, they can migrate to the surface and react with water, oxygen, and other molecules at the interface. Electrons can react with oxygen to form the oxidizing O_2_˙^−^ superoxide radical:

2 O_2_ + e^−^ → O_2_˙^−^

However, O_2_˙^−^ is so reactive and can oxidize molecules and transforms itself into HO˙ *via* the following reactions:

3 O_2_˙^−^ + H^+^ → HO_2_˙

4 HO_2_˙ → → H_2_O_2_ + O_2_

5 H_2_O_2_ + O_2_˙^−^ → → HO˙ + O_2_ + HO^−^

H_2_O_2_ + *hν* → → 2HO˙

While, at the HOMO level, the holes may react with HO^−^ (or H_2_O) to produce oxidative HO˙:

6 H_2_O + h^+^ → → HO˙ + H^+^

**Fig. 16:**
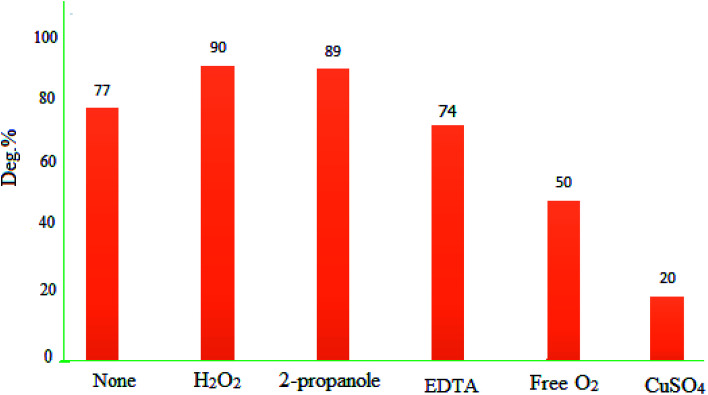
Effects of some hole scavengers on the photodegradation of MB (40 mL of 8 ppm MB solution, 0.005 mmol of hole scavenger, pH 7, and MgZnO@SiO_2_-tetrazine: 5 mg).

To identify the responsible species for the photodegradation activity of MgZnO@SiO_2_-tetrazine, some tests were performed with Cu^2+^ and 2-propanol, as electron and hole scavengers, respectively. Data showed that presence of Cu^2+^ slowed down the degradation kinetics. Cu^2+^ reacted with electrons to yield Cu^+^, and this reaction was in competition with reaction (2). The presence of Cu^2+^ may lead to diminishing of the generated O_2_˙^−^ and, therefore, decreasing of the degradation kinetics. The photodegradation efficiency of MB was decreased from 77 to 22% in the presence of Cu^2+^ (0.01 M) after 40 min under UV-vis light irradiation. Also, in the absence of O_2_, the reactivity of MgZnO@SiO_2_-tetrazine for MB degradation was diminished and deg% decreased from 77 to 50% after 40 min irradiation.^38^ Moreover, other experiments were performed in the presence of 2-propanol to scavenge the holes and HO˙ radicals. The experiments in the presence of O_2_ and 2-propanol did not show any significant decrease of photodegradation. Additional experiments were planned with other scavengers such as EDTA and H_2_O_2_ as holes (h^+^), and HO˙ scavengers, respectively. Fig. 16 shows that EDTA and H_2_O_2_ did not inhibit the photocatalytic activity of MgZnO@SiO_2_-tetrazine. All these experiments proved that photodegradation of MB by MgZnO@SiO_2_-tetrazine was mainly caused by O_2_˙^−^.

**Fig. 17:**
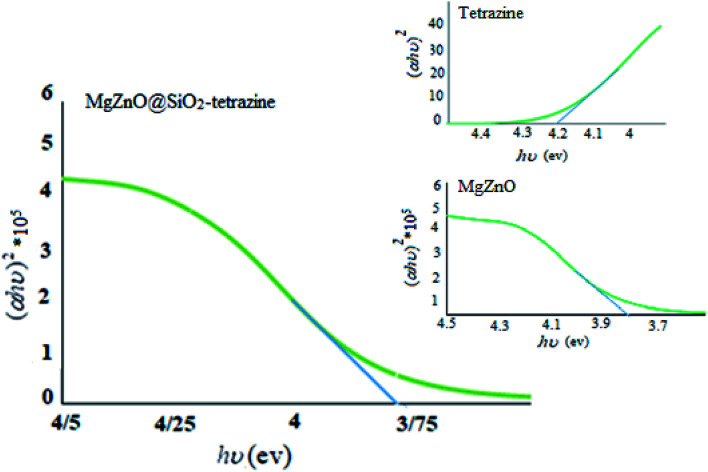
Plots of (*αhυ*)^2^*vs.* photon energy of tetrazine, MgZnO, and MgZnO@SiO_2_-tetrazine.

To assign the optical band gaps, the optical absorbance measurements were planed at room temperature and the absorption spectra of tetrazine, MgZnO, and MgZnO@SiO_2_-tetrazine were attained. Absorbance spectra showed an absorption peak around 282 nm for tetrazine, 335 nm for MgZnO, and 332 nm for MgZnO@SiO_2_-tetrazine. To compute the direct band gap, the Tauc relation was utilized:^39^(*αhυ*)^2^ = *A*(*hυ* − *E*_g_)where *α* is the absorption coefficient and “*A*” is a constant. An extrapolation of the linear region of the plot of (*αhυ*)^2^*vs. hν* gave the values of optical band gaps as 4.2, 3.82, and 3.75 eV for tetrazine, MgZnO, and MgZnO@SiO_2_-tetrazine, respectively, as shown in Fig. 17.^40,41^ The Taugh plots indicated that the optical band gap of MgZnO@SiO_2_-tetrazine is relatively smaller than that of MgZnO and tetrazine. It means that the electrons may transfer to the conductive band with lower energies and the nanocatalyst could absorb photons with longer wavelengths. Therefore, the absorption band was expanded to the visible region of spectrum and the absorption edge shifted to longer wavelengths. It means the optical absorption has been increased.^42–45^ The photocatalytic activity and, therefore, quantum efficiency of MgZnO@SiO_2_-tetrazine was almost consistent with the Taugh plots.^46,47^ Thus, it can be envisaged that the photocatalytic activity is closely related to the optical absorption. As the optical absorption is increased, the photocatalytic activity was also enhanced.

**Scheme 1:**
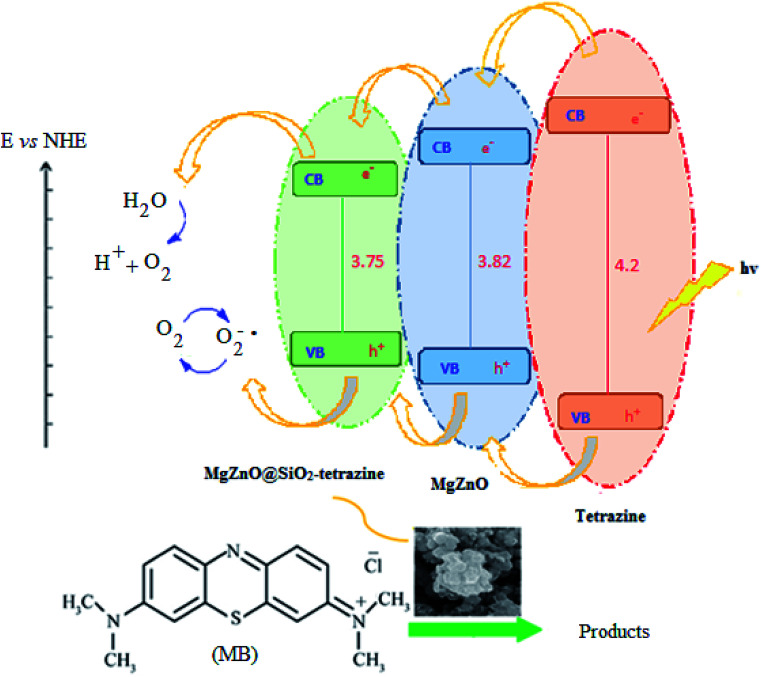
Proposed photocatalysis mechanism for MgZnO@SiO_2_-tetrazine.

A plausible photocatalytic mechanism with charge separation in MgZnO@SiO_2_-tetrazine involving both hole oxidizing water and electron reducing oxygen is proposed in Scheme 1. O_2_ produced at the valence band (VB) can taking part in the O_2_ reduction at the conduction band (CB). Moreover, the generated electrons and holes contributed to the degradation process.

## 3. Experimental

### 3.1. Materials and methods

Starting solvents and materials were purchased from Sigma-Aldrich, Fluka, and Merck, and were utilized as received without further purification. A high-pressure mercury vapor lamp (NAVIFLUX, 400 W, Berlin, NARVA) with lamp operating current of 3.25 A and a nominal voltage of 230 V was employed for the photocatalytic experiments. Morphologies of the synthesized nanomaterials were studied by a Mira 3-XMU field emission scanning electron microscope (FESEM). Fourier transform infrared (FT-IR) spectra were performed on a Shimadzu 8700 Fourier transform spectrophotometer in the range of 400–4000 cm^−1^ with KBr pellets. UV-visible spectra were recorded by using a Photonix UV-visible array spectrophotometer. Elemental analyses were done by a Thermo Finnigan Flash-1112EA microanalyzer. X-ray diffraction patterns (XRD) were acquired on an Xpert MPD diffractometer with Cu K_α_ radiation at 30 mA and 40 keV under the scanning rate of 3° min^−1^ in the 2*θ* domain from 5–80°. Thermogravimetric analyses were performed on a TGA 92 Setaram at a rate of 10 °C min^−1^. The chemical composition of the prepared materials was determined by using an inductively coupled plasma spectrometer (ICP-MS; model VARIAN VISTA-PRO). Diffuse reflection-transmittance (DRS-DTS) spectra were recorded on an Avaspec-2048-TEC.

### 3.2. Preparation of Mg_0.007_Zn_0.093_O nanoparticles (denoted as MgZnO)

Doped MgZnO was prepared by the sol–gel process. A methanol solution of zinc acetate dihydrate (50 mL, 0.093 M) was blended with another methanol solution of magnesium acetate tetrahydrate (50 mL, 0.007 M). Then, 10 mL of ethylene glycol (C_6_H_6_O_2_) was added to the final solution and stirred for 30 min. After that, the solution was warmed up gradually to 70 °C with continuous stirring until a clear white gel was developed. The obtained gel was kept-up at room temperature for 24 h and, then, was heated to 250 °C for 6 h. Eventually, the solid product was well grounded and heated to 650 °C for 10 h to obtain a white color Mg_0.007_Zn_0.093_O (MgZnO) nano-powder. A similar procedure was followed for the preparation of other Mg_1−*x*_Zn_*x*_O metal oxides by changing the respective proportion of the reactants.

### 3.3. Surface modification of MgZnO by anchoring of (3-chloropropyl)triethoxysilane

**Fig. 18:**
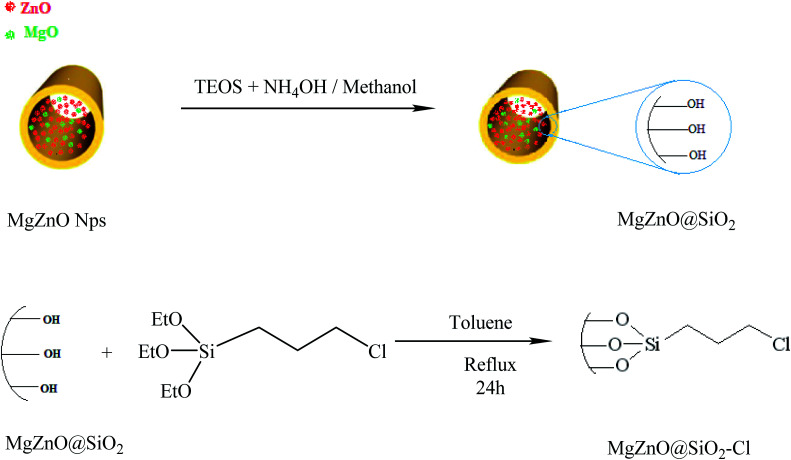
A schematic representation for the preparation of MgZnO@SiO_2_–Cl.

The silica-coated MgZnO@SiO_2_ was prepared through a simple method.^48^ Briefly, 1 g of MgZnO nanoparticles was dispersed in a mixture of ethanol (60 mL), deionized water (20 mL), and concentrated ammonia (28 wt%, 2 mL) under ultrasonic for 30 min. To this solution, 0.43 mL of tetraethylorthosilicate (TEOS) was added drop-wise. The obtained product was collected after 16 h mixing and washed with ethanol and deionized water. Finally, the silica coated MgZnO@SiO_2_ was dried under air at 60 °C for 8 h. 0.5 g of MgZnO was dispersed into anhydrous toluene (20 mL) and 0.7 mL of (3-chloropropyl)triethoxysilane (CPTS) was adjoined to this suspension and, then, the mixture was refluxed for 24 h. Thereafter, the obtained precipitate was dried at room temperature (Fig. 18).

### 3.4. Preparation of 4-((6-chloro-1,2,4,5-tetrazine-3-yl)oxy)butyl propylcarbamate

**Fig. 19:**
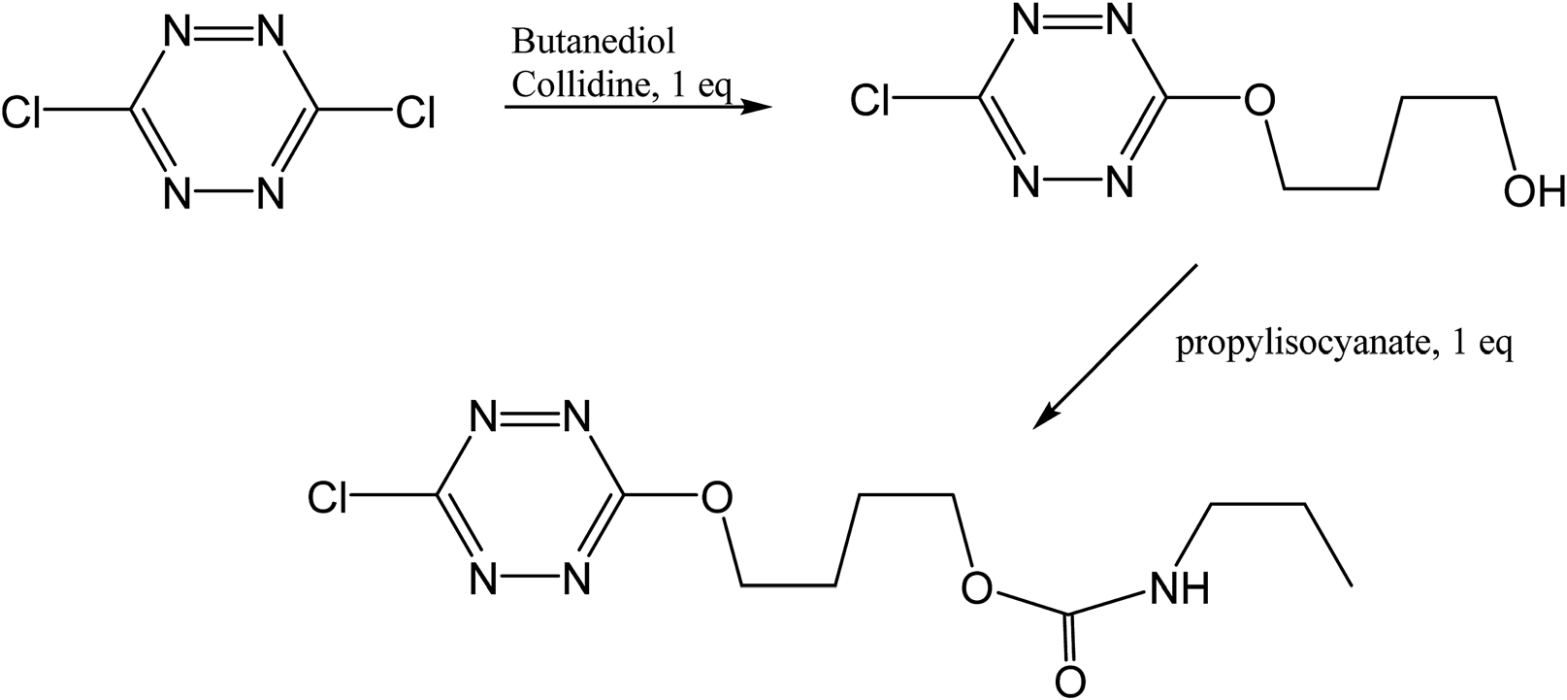
Synthetic scheme for the preparation of “tetrazine”.

4-((6-chloro-1,2,4,5-tetrazine-3-yl)oxy)butyl propylcarbamate was prepared from the reaction of 4-((6-chloro-1,2,4,5-tetrazine-3-yl)oxy)butanol^49^ with propylisocyanate according to Fig. 19. The experimental conditions were completely standard as described before.^50^

### 3.5. Immobilization of tetrazine onto MgZnO@SiO_2_–Cl

**Fig. 20:**
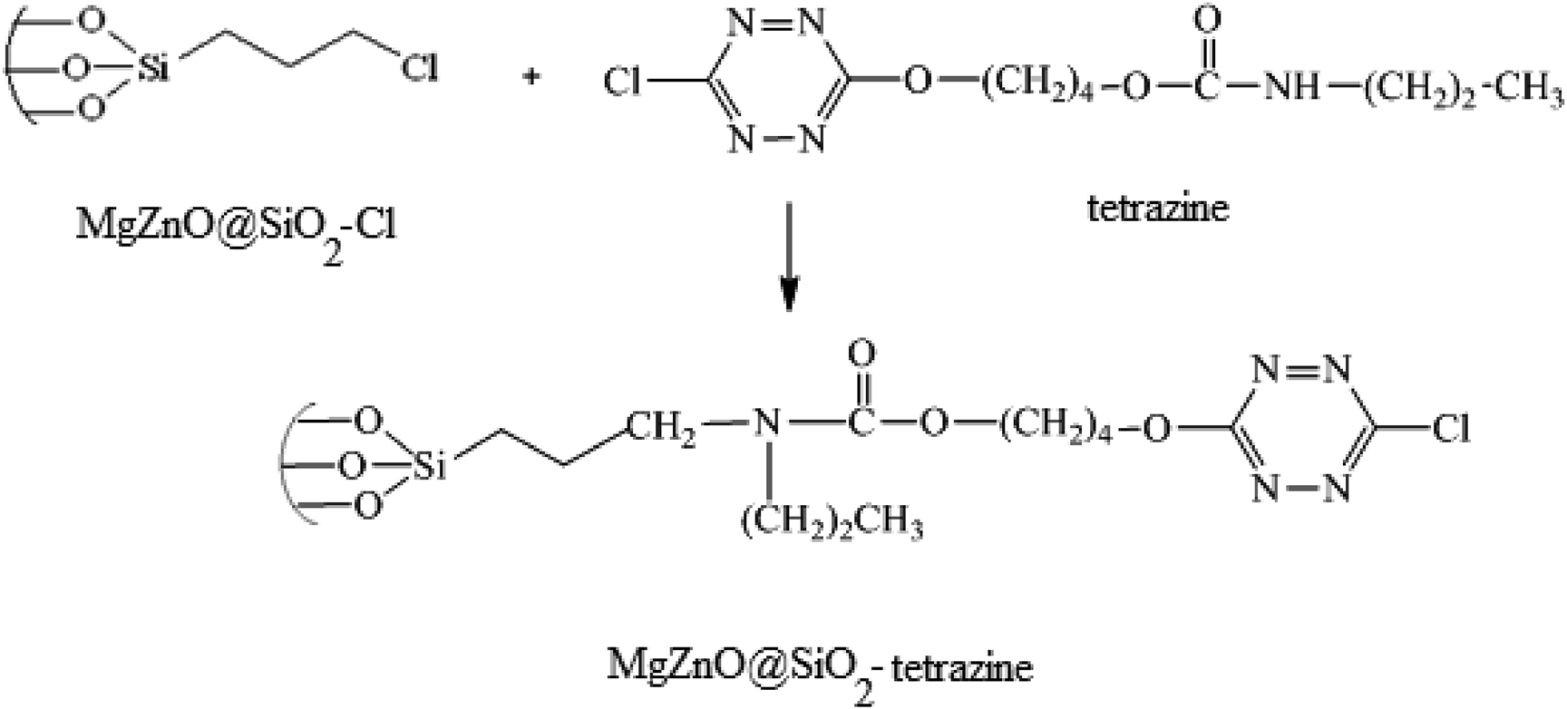
A schematic for the preparation of MgZnO@SiO_2_-tetrazine.

In the next step, for the immobilization of 4-((6-chloro-1,2,4,5-tetrazine-3-yl)oxy)butyl propylcarbamate (abbreviated as tetrazine) onto MgZnO@SiO_2_–Cl, 0.4 g of MgZnO@SiO_2_–Cl was dispersed in 20 mL of dichloromethane under sonication. Then, a solution containing 0.2 g of tetrazine in 10 mL of dichloromethane was mixed with the above solution and refluxed for 24 h. Finally, the resultant precipitate was separated, washed thoroughly with dichloromethane, and dried at room temperature. Fig. 20 displays a schematic for the preparation of MgZnO@SiO_2_-tetrazine.

### 3.6. Photochemical procedure

The photocatalytic activity of the heterogeneous MgZnO@SiO_2_-tetrazine nanoparticles was assessed by suspending the catalyst into a solution of MB inside a simple hand-made Pyrex reactor equipped with a water circulating system at ambient temperature. First, the heterogeneous solution was stirred for 30 min in the absence of light to obtain the adsorption–desorption equilibrium of the dye molecules onto the nanocatalyst surface. Afterward, the photoreactor was adjusted at a distance of 8 cm from the light source and illuminated at 24 ± 1 °C for the required time. Diminishing of the dye concentration was monitored by a UV-vis spectrophotometer. The degradation efficiency of the analyte was calculated using eqn (7):

7 Degradation yield (%) = [(*A*_0_ − *A*) − *A*_0_] × 100

where *A*_0_ and *A* are absorbances of the analyte before and after irradiation, which can be easily correlated to the final (*C*) and initial (*C*_0_) concentrations of the dye, respectively.

## 4. Conclusions

MgZnO@SiO_2_-tetrazine nanoparticles were synthesized, characterized, and utilized as an effective heterogeneous photocatalyst for the photodegradation of MB and ciprofloxacin under the irradiation of a high-pressure mercury lamp in the UV-vis region. The effects of several parameters on the photodegradation of MB had been investigated. Enhancing the photocatalyst amount up to 0.02 g at 40 mL of MB solution increased the photodegradation efficacy. However, the efficiency was diminished when a higher concentration was used; perhaps due to the aggregation of the solid nanoparticles. The optimum pH was about 9; whereas a higher pH reduced the photodegradation efficiency. High MB concentration up to 3.5 mg L^−1^ enhanced photodegradation; however, beyond a dye concentration of 3.5 ppm, the photocatalytic degradation was alleviated. Moreover, the optical absorbance measurements were done at room temperature and the optical band gaps of 4.2, 3.82, and 3.75 eV were attained for tetrazine, MgZnO, and MgZnO@SiO_2_-tetrazine. The catalytic stability of the recycled MgZnO@SiO_2_-tetrazine was evaluated, and a high stability and reusability was attained. Pseudo-first-order kinetics with a high rate constant (0.068 min^−1^) were obtained for MB degradation. Possible photocatalytic mechanism, which contributes to the oxidation of MB may include both reduction of oxygen with electrons and holes oxidation of water molecules.

## Conflicts of interest

There are no conflicts to declare.

## Supplementary Material
